# Health Effects of Happiness in China

**DOI:** 10.3390/ijerph19116686

**Published:** 2022-05-30

**Authors:** Weiwei Wang, Yan Sun, Yong Chen, Ya Bu, Gen Li

**Affiliations:** 1 School of Economics and Management, Jiangsu University of Science and Technology, Zhenjiang 212000, China; wangweiwei@just.edu.cn (W.W.); ybu@just.edu.cn (Y.B.); ligen_78@163.com (G.L.); 2 College of Humanities and Social Sciences, Jiangsu University of Science and Technology, Zhenjiang 212000, China; saadiya99@126.com; 3 School of Civil Engineering and Architecture, Zhejiang University of Science and Technology, Hangzhou 310023, China

**Keywords:** happiness, self-rated health, aging, rural, mediate

## Abstract

The demand for improving health status of Chinese residents is growing with the rapid economic development. Happiness, which could be improved by some brief, self-administered, and cost-effective interventions, is reported to be associated with mortality, longevity, and self-rated health. Therefore, it is essential to assess the effect of happiness on health in China. Using data from the Chinese General Social Survey 2017, the present study explored the effect of happiness on health among Chinese residents after controlling for demographic variables, socioeconomic factors, social relationships, locations, and insurance plan. The happiness effect across subsamples by age and resident type and the mediator role of happiness were also evaluated. Based on an ordered probit regression model, we found that the effect of happiness on health was significantly positive in full sample and all subsamples. Using a structural equation model, we demonstrated that happiness could partially mediate the relationship between socioeconomic factors, social relationships factors, and health. Our data supplement the existing literature on the relationship between happiness and health and provide evidence for policymakers and stakeholders focusing on happiness as a health strategy to improve overall societal wellbeing.

## 1. Introduction

With an unprecedented economic growth in China over the past half-century, health issues have gained widespread attention from Chinese citizens and policymakers. The Chinese government is increasingly recognizing the importance of nationwide health and is emphasizing the priority of the health status of residents. In 2009, China launched a major health care reform aiming to provide equal access to basic health care for all citizens with reasonable quality and financial risk protection [1]. In 2016, the Communist Party Central Committee and the State Council of China released the Outline of Healthy China 2030 Planning, which declared that the construction of Healthy China was a keystone for building a prosperous society. Efforts and funding to improve nationwide health have largely increased. Subsidies to primary health institutions have increased from USD 2.8 billion in 2008 to USD 20.3 billion in 2015. In addition, the status of health facilities, medical technology, health human resource, coverage of social medical insurance, and access to health services have continually improved. Moreover, China is becoming a graying society with adults older than 60 composing nearly one third of the population by the year 2050 [2]. Considering that the prevalence of chronic illness increases with advancing age, the demand for researching the factors that influence the health status among Chinese residents is growing rapidly.

Subjective well-being, referred to happiness or satisfaction, has increasingly gained attention from researchers in recent decades [3]. Research has revealed that happier people tend to live longer and have better health, better social relationships, higher work productivity, and good citizenship [4]. The improvement of happiness of the population is emerging as a key societal aspiration. Happiness has been proposed to be an indicator of the progress of society [3]. Efforts to assess and improve happiness might help countries to understand and improve what really matters to people. Some positive, brief, self-administered, and cost-effective intervention strategies that could increase happiness have been demonstrated in a rapidly growing literature [3]. The interventions include cultivating gratitude [5,6,7,8,9], performing acts of kindness [8], visualizing one’s best possible self in the future [5,10,11], writing about one’s positive experiences or sharing these positive experiences with others [12,13], using one’s character strengths in new ways [9], and so on.

The positive effect of happiness on health has attracted extensive interest from many fields of social research. Hundreds of longitudinal studies have found that happier individuals live longer [14,15,16]. The same result has been replicated in studies with apes, in which happier orangutans live longer [17]. A cotwin control study over a period of 70 years demonstrated that a one standard derivation increased in positive affect was associated with a reduction of 9% in mortality risk after adjusting for the number of illnesses and medication and cognitive composite score [18]. Studies in twins are considered to be good indicators of causal associations due to the exclusion of cofounders caused by genetics and environmental factors. A meta-analysis of 26 prospective studies on initially healthy populations and 28 studies on disease populations (with established diseases such as HIV/AIDS) revealed that happiness was associated with a reduced mortality in both healthy people and patients [19]. Davidson et al. performed a large prospective study with 10 years of follow-up and found that positive affect functioned as a protective factor against cardiovascular diseases [20]. In addition, a study that enrolled a representative sample of 817 residents from Italy showed that happiness was strongly correlated with perceived health after adjusting for a number of relevant socioeconomic factors [21]. Although there is extensive progress about the protective role of happiness on health, most studies have only focused on developed countries or regions. Furthermore, Liu et al., (2016) recently reported that although chronic illness caused unhappiness, unhappiness itself had no direct effect on mortality [22]. They found that there were no differences in the overall death rate between those who reported to be unhappy and those who did not after adjusting for diseases and lifestyle. Thus, further research is needed to explore the role of happiness on health across different countries and culture with serial related variables controlled.

The mechanisms of the positive effect of happiness on health have already been proposed. Findings from medicine and psychology have suggested that psychosomatization is the main transmission mechanism connecting happiness to health [21]. A negative emotional response could significantly influence the function of the autonomic nervous system, which might activate physiological reactions that cause cumulative detrimental effects on health [23]. Negative psychological states or traits, such as depression, anxiety, and psychological distress, were reported to be associated with the increased risk of Type 2 diabetes, coronary heart disease, disability, and total mortality [24,25,26,27,28,29]. Happiness may exert its beneficial effects on physical health through preventing the activation of these physiological reactions [30]. Indeed, happier individuals were found to have stronger immune and cardiovascular systems, as well as better cortisol and blood pressure parameters [31,32,33]. Extensive prospective observational studies showed that positive psychological well-being was associated with reduced mortality in both the healthy population and the diseased population, and the protective effects of positive psychological well-being were independent of negative affect [19]. Healthy behaviors are an additional link between happiness and health: happy people are more likely to engage in regular physical exercise, to watch their weight, and to avoid unhealthy behaviors [23,34,35]. Therefore, reverse cause–effect relationship makes it hard to conclude on the specific happiness effect.

Empirical evidence points that there are similar causal mechanisms underlying both happiness and health. In the last decades, the socioeconomic determinants have been extensively studied in happiness and health separately. Studies have revealed that residents with higher socioeconomic status are much healthier and experience lower mortality rates than those with a lower socioeconomic status [36,37]. Differences in self-rated health among residents with different income have also been reported after adjusting for age, gender, race, and marital status [38]. In addition, a survey among elderly Palestinian women showed that women with higher income than the national average monthly income were more likely to have better self-rated health than those with poorer households [39]. Meanwhile, it was demonstrated that happiness was strongly affected by relative income as well as absolute income [40,41]. A spline regression model which enrolled 1.7 million individuals revealed that income was fairly linearly associated with happiness. However, this relationship ended around the annual income of USD 60,000–75,000 [42]. The correlation between income and health is similar to that between happiness and income, which is quite steep for lower incomes, becomes less steep for higher incomes, and satiates after a certain threshold [43,44,45]. After the specific threshold, relative income and social factors take over the crucial causal role for happiness and health. Relative deprivation and frustrated aspirations create stress, which are sometimes sources of chronic and psychosomatic diseases, as well as persistent unhappiness [43]. Social relationships within the family, with friends and other members of the community in which the individual chooses or is born into, are another important source of happiness and health [43]. Holt-Lunstad et al. (2010) performed a meta-analysis and demonstrated that social relationships were associated with the risk of mortality [46]. Socializing with family and friends has also been reported to be positively associated with happiness [47,48]. Moreover, a large body of studies have shown that social support could serve as a predictor for better physical and mental health [49,50]. Social relationships deterioration could contribute to unhappiness and health degradation.

Happiness, which is valued by everyone, seems to be a cost-effective strategy for improving health [14,15,17,18,19,21]. In China, the government and public also pay more and more attention to happiness, the report of the 19th National Congress put forward the goal to “make people’s happiness more substantial, more secure and more sustainable”, and many cities have put forward the goal of building a “happiness city”. People’s happiness has become an important aspect for policymakers to consider in China. In addition, the demand for cost-effective interventions to increase health status among Chinese residents is increasing. There is a growing body of research that suggests a relationship between happiness and health, and it is meaningful to use a large sample to study the impact of happiness on health in the largest developing country in the world. Moreover, happiness and health share similar determinants, such as socioeconomic factors and social relationships, which may affect the results of this correlation. For the above reasons, this study had the following aims: (1) to assess the effect of happiness on health after controlling for potential confounding factors; (2) to evaluate the happiness effect across subsamples by age and resident type considering that there is a difference in health of the young and the old, rural and urban residents (described in the discussion section); (3) to explore whether socioeconomic factors and social relationships could influence health directly as well as indirectly through happiness.

## 2. Methods

### 2.1. Data Sources

In the present study, the data were from the Chinese General Social Survey (CGSS) conducted in 2017, which is an open access, nation-wide, comprehensive, large-scale social survey project with the aim to reflect the transition of economics, politics, society, and culture in China. The CGSS use a multistage stratified probability-proportional-to-size (PPS) random sampling, the way of investigation is face-to-face interview, and the language of survey is Chinese. In addition, CGSS can be used for academic research free of charge. There were more than 10,000 people investigated in each round. Moreover, more than 2000 research articles have been published based on CGSS data by 2018. The datasets in 2017 contain a total of 12,582 valid questionnaires from 28 provinces, municipalities, and autonomous regions of China. After the missing values and invalid answers were removed from the samples, a total of 11,009 valid samples were included in this study.

### 2.2. Variables

A description of the variables is shown in Table 1. Self-rated health has been proved as a valid and predictive health indicator in many studies [51,52,53]. It has been demonstrated that self-rated health is strongly associated with mortality [52]. A review of 27 communities concluded that self-rated health did a fairly good job even after controlling for the objective health indicators [21]. Thus, the health status of residents was measured by self-rated health in this study. In the CGSS 2017, respondents were asked to rate their health status using a five-point Likert scale ranging from 1–5 (very unhealthy = 1 to very healthy = 5). A large body of literature has demonstrated the validity, reliability, and comparability of using single-question answers to evaluate happiness [54,55]. Thus, happiness was measured by using responses to the question, “Generally speaking, how do you personally feel about your life?” on a five-point Likert scale, where very unhappy = 1, neither happy nor unhappy = 3, and very happy = 5.

We selected control variables that might be both related with self-rated health and subjective well-being. The natural logarithm of annual household income, self-rated individual social class, self-rated household economic level in the place of residence were included in our study as the indicators of socioeconomic variables. Friend interaction, meeting relatives, and physical exercise were used to indicate physical exercise and social relationships. In addition, we controlled for demographic variables (gender, age, marital status, and education), insurance plan, resident type, and a full set of province dummy variables in the ordered probit regression. The resident type was identified by the household registration type, which is a dummy variable used to distinguish between rural and urban residents. Regarding insurance plan, the most widely subscribed insurance plan in China is the basic health insurance (BHI) plan. A personal commercial medical insurance (PCMI) plan targeted at those who are willing and able to afford PCMI payments was also considered.

### 2.3. Model Selection

In this study, an ordered probit model (Equation (1)) was used to investigate the effect of happiness on health:

y*_i_ = *α* + *β_i_X_i_* + *ε* (1)

where y*_i_ denotes individual self-rated health and the vector *X* includes happiness, socioeconomic factors, social relationships, and other control variables. Additionally, the variance inflation factors (VIF) of all variables were analyzed, showing that all values were lower than 2. This was far below the critical value of 10, indicating no significant multicollinearity in the model. To analyze the happiness effect across subsamples by age and by resident type, the age threshold was set at 60 years old according to the standard of the World Health Organization, which considers those aged 60 or over as the old and those under this age as the young. As for the resident type, rural and urban resident were studied. Then, we selected the ordinary least squares (OLS) regression, binary probit regression, and instrumental variable of ordered probit (IVoprobit) method to conduct the robustness test.

To explore whether socioeconomic factors and social relationships could directly influence health as well as indirectly through happiness, a structural equation model (SEM) was performed. The relevant matrix equations are as follows:

*X* = *Λ_X_ξ* + *δ* (2)

*Y* = *Λ_Y_η* + *ε* (3)

*η* = *Bη* + *Γξ* + *ζ* (4)

where *X* and *Y* refer to the observational variables vector of exogenous and endogenous latent variables, respectively; *ξ* and *η* refer to the vector of exogenous and endogenous latent variables, respectively; *Λ_X_* and *Λ_Y_* are the factor-loading matrices; *B* denotes the relationship between the endogenous latent variables; *Γ* represents the effect of exogenous latent variable on the endogenous latent variable; *δ*, *ε*, and *ζ* are the residual item. The structural model was tested with the maximum likelihood estimation. To assess the fit of the model to the data, indexes including chi-square divided by degrees of freedom (χ^2^/DF), the Tucker–Lewis index (TLI), the comparative fit index (CFI), the root mean square error of approximation (RMSEA), and the incremental fit index (IFI) were calculated.

### 2.4. Statistical Analysis

A Student’s *t*-test was used to compare the levels of self-rated health between two independent groups. If more than two groups were compared, a one-way analysis of variance was used. An ordered probit model was used to investigate the effect of happiness on health, the ordinary least squares (OLS) regression, binary probit regression, and instrumental variable of ordered probit (IVoprobit) method were used to conduct the robustness test. All basic statistics and regressions were performed with STATA 15.0 (StataCorp, College Station, TX, USA). Finally, a structural equation model (SEM) analysis was used to study the mediator role of happiness using IBM Amos 24.0 (Amos Development Corporation, Chicago, IL, USA).

## 3. Results

### 3.1. Descriptive Statistics

**Figure 1:**
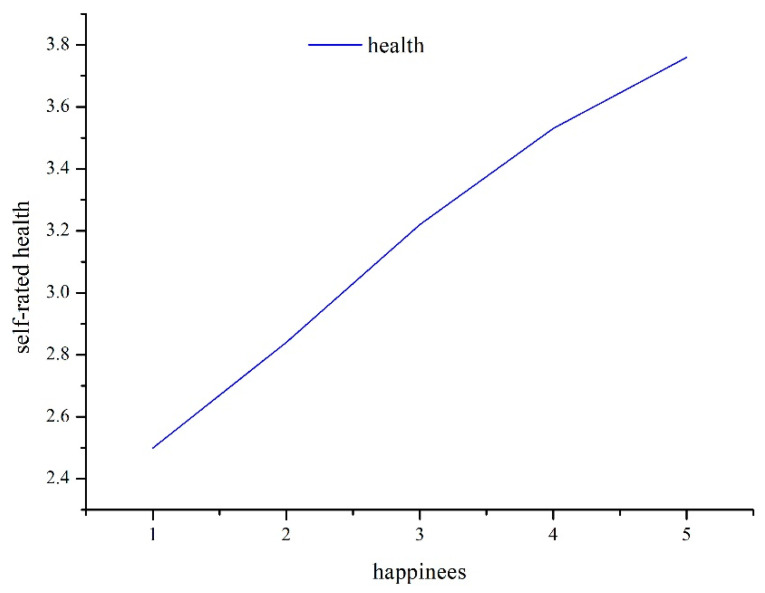
The change of self-rated health with happiness.

The mean and standard deviation (SD) of all variables are shown in Table 1. The distribution and percentage of all variables, as well as the average levels of self-rated health in different variable groups, are shown in Table 2. The mean age was 49.97, the elderly people, whose age is 60 or above, accounted for 34.3%. The female and unmarried accounted for 52.4% and 11.3% of total samples, respectively. The proportion of people with higher education and above was only 19.2%. The results showed that the average score of self-rated health was 3.46, and that 53.4% of the respondents reported that they were healthy. The mean score of happiness was 3.85, which was higher than that in 2005 and 2010 [56]. There were 77.8% of residents saying that they are happy, and they tended to have a higher self-rated health than unhappy residents (3.58 vs. 3.04, *p* < 0.001). We could also see that the self-rated health increased along with the increase of happiness (Figure 1).

**Figure 2:**
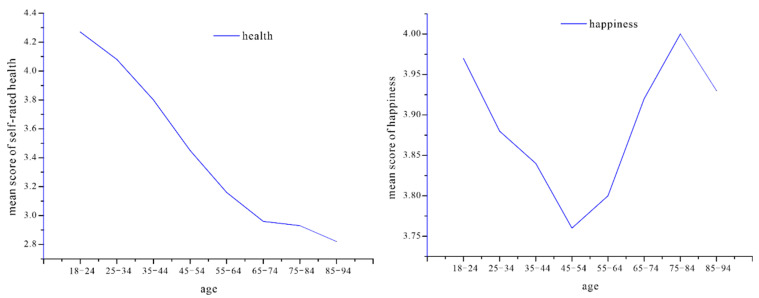
The change of self-rated health and happiness with aging.

It was obvious that the self-rated health decreased significantly along with the increase of age (*p* < 0.001), which is consistent with the notion that age is an important factor influencing the health status of residents (Figure 2). Therefore, we performed the regression analysis separately across subsamples by age in the following section. However, the relationship between happiness and health revealed a V-shaped model with the lowest levels of happiness in the age group 45–54 years (Figure 2). It was similar to the U-shaped model conducted in high-income, English-speaking countries [57]. In addition, the urban respondents reported a higher self-rated health than the rural respondents (3.60 vs. 3.35, *p* < 0.001). Considering that the unbalance in health and happiness between rural and urban residents were reported in previous studies [56,58], we estimated the happiness effect for rural and urban subsamples in further regressions. Data also showed that there was a clear monotonic increase in the self-rated health score with the rise of the education level, and the differences were statistically significant (*p* < 0.001).

### 3.2. Full Sample Analysis

An ordered probit model was used to evaluate the impact of happiness on health, and the robustness of the regression was maintained by adding control variables sequentially. In addition, a full set of province dummy variables were controlled in all models. The regression results of the full sample analysis are shown in Table 3. The first column (model 1) presents the regression results obtained from a parsimonious model that only control respondents’ age, gender, marital status, education. Model 2 expands the variables by including measures of income, social class, and economic level as indicators for socioeconomic factors. Model 3 adds physical exercise and social relationships (physical exercise, friend interaction, meeting relatives) into the regression. The specification reported in Model 4 adds insurance plan (BHI, PCMI).

The results in Table 3 confirmed that being happier represented a gain in the health of individuals. Looking at the correlation between happiness and health, there are other factors associated with both health and happiness, such as socioeconomic factors and social relationships. Therefore, it is unsurprising that the size of the happiness coefficient is reduced when socioeconomic factors and social relationships are controlled (from model 1 to model 3). The marginal effect of happiness on self-rated health is shown in Table 4; we can see that the probability of reporting being unhealthy decreases and reporting being healthy increases for happier people. This further illustrates that happiness has a significant positive effect on self-rated health.

### 3.3. Subsample Analysis

Much of the unbalance in China is a rural–urban phenomenon. If the unbalance affects health and happiness to a different extent, the effect of happiness on health may be influenced. In addition, the correlation between happiness and age (U-shaped) is different from that between health and age (linearly declining) [59]. Therefore, we evaluated the effect of happiness on health separately in rural and urban subsamples and in the young and the old subsamples to study whether there were differences in the impact effects. As shown in Table 5, happiness kept a significantly positive impact on health across all subsamples after adjusting for demographic variables, socioeconomic factors, social relationships, insurance plan, and locations. In addition, the marginal effects of happiness on self-rated health in the subsamples were similar to those of the full sample analysis (Table 6).

### 3.4. Robustness Check

Self-rated health was proposed to be an outcome as a function of happiness. However, self-rated health might be seen as another variable contributing to happiness. In order to solve the endogeneity, the spouse’s education was used as the instrumental variable. There is correlation between a spouse’s education and happiness, but spouse’s education is not likely to influence self-rated health directly. Therefore, we think the spouse’s education might be an appropriate instrumental variable to try to solve this endogeneity. The F-statistic of the instrumental variable was 11.07, greater than 10, indicating that instrument variable was related to the endogenous variables with strong significance; the results are shown in Table 7. Then, we used an ordinary least squares (OLS) regression and a binary probit regression to test the robustness of results. The results of IVoprobit, OLS, and binary probit were consistent with those of the ordered probit regression. This further proved that happiness had a significant positive impact on self-rated health.

### 3.5. The Mediator Role of Happiness

**Figure 3:**
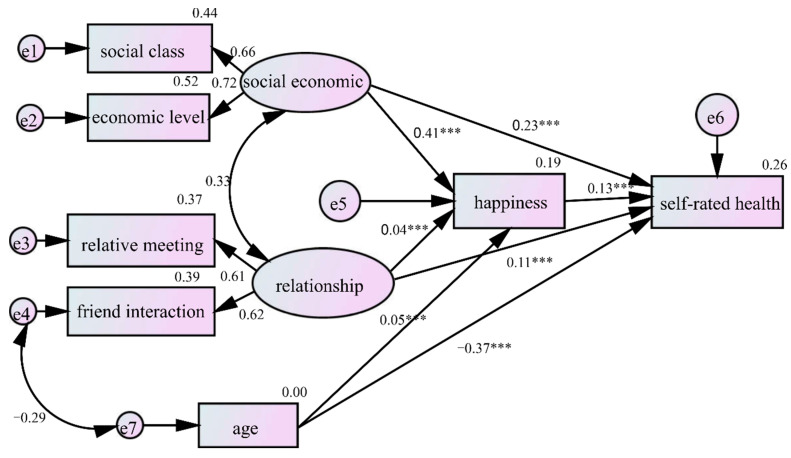
Results of the structural model (standardized outcome). *** *p* < 0.01.

The fit indices of the structure equation model are listed in Table 8; the acceptable fit criteria were set in accordance with previous studies [59,60]. The chi-square statistic was reported, but not taken into account because the large number of observations mad it inadequate as a fit estimate. Except for the chi-square, all fit indices indicated that this model retained an acceptable fit to the data. As shown in Figure 3, this model explains 26% of the variance in self-rated health. In addition to the direct effect, socioeconomic factors, social relationship, and age could exert their effects on self-rated health indirectly through happiness. The major results of this model are presented in Table 9.

## 4. Discussion

To explore the effect of happiness on health among Chinese residents, we controlled a series of factors that could influence both happiness and health, including age, gender, marital status, socioeconomic factors (income, social class, and economic level), physical exercise, social relationships (friend interaction and meeting relatives), whether rural or urban residents, provinces, and insurance plan. After adding the control variables sequentially, we found that the effect of happiness on health remained significantly positive, and there was some reduction of the coefficient of happiness after the addition of socioeconomic factors and social relationships. Socioeconomic factors and social relationships could affect both happiness and health and the effect of happiness on health may be exaggerated without considering these factors. Unfortunately, we could not include all factors that influence both happiness and health due to the limited knowledge about happiness and health determinants, as well as the datasets used in this present study lacking some data about known variables for happiness and health, for example, the environmental factors. In addition, self-rated health might be seen as another variable contributing to happiness. Then, we selected the instrumental variable of ordered probit (IVoprobit) method to try to solve the latent endogeneity; our result that happiness has a significantly positive effect on self-rated health would be stable if the instrumental variable was appropriate. Although our data about the happiness effect should be interpreted with caution, our efforts to figure out the happiness effect may provide support for further study.

China has experienced rapid urbanization since the economic reforms and opening. Urbanization has brought a substantial and complex influence on the health and happiness of Chinese people [56,61,62,63,64]. On the one hand, people in urban areas generally enjoy better health care and living standards than their rural counterparts. On the other hand, residents in urban areas tend to experience more environmental pollution, sedentary lifestyles, and life stresses. The disparities in happiness and health determinants between rural and urban residents motivated us to further investigate the happiness effect on health among residents in the two regions. Considering that socioeconomic factors and social relationships could affect both happiness and health, we hypothesized that happiness might serve as a proxy of the effect of socioeconomic factors and social relationships on health. Using the SEM, we found that happiness could partially mediate the effects of social relationships, socioeconomic factors, and age on health. Thus, individuals with a higher socioeconomic status and better social relationships tended to be happier, hence prone to have a better self-rated health.

### Limitation

Most of the variables in this article relied on self-reported measures, which may lead to some measurement deviations, especially for the self-reported health that is the dependent variable in this study. Although the self-reported health could reflect a person’s actual health, some deviations might inevitably emerge; for example, the mood of the respondents during the survey may have affected their answer about their health. However, self-reported health and objective measures should have a similar trend that leads to a strong correlation between these two measures. If these two different measures have the same constructs, the difference between self-rated and objective measures could be regarded as the measurement error of the objectively measured health status. As long as this measurement error is not related to the explanatory variable, our estimated results will be consistent. If the measurement error is related to the explanatory variable for common aspects of the type of measurement, our estimated results will not be consistent. The instrumental variable (IV) method can solve this potential endogeneity if the instrumental variable is appropriate.

## 5. Policy Implications

Our results showed that the effect of happiness on health was significantly positive. This further proved the importance of happiness in residents’ daily life and provided evidence for policymakers and stakeholders focusing on happiness as a health strategy. Our results showed that there was a partial mediation effect in the relationship between happiness and self-rated health through socioeconomic factors, social relationships factors, and age. In addition to the direct effect, socioeconomic factors, social relationship, and age could exert their effects on self-rated health indirectly through happiness. This provides evidence for policymakers when creating policy to improve happiness and health at the same time. Our results showed that age has a significant negative impact on health. China is becoming a graying society with adults older than 60 composing nearly one third of the population by the year 2050 [2]. Considering that the prevalence of chronic illness increases with advancing age and the issue of maintaining good health at an advanced age is growing in importance, the research on the influencing factors of subjective well-being of the elderly should increase to improve overall societal wellbeing.

## 6. Conclusions

This article evaluated the association between happiness and self-rated health among Chinese residents. Our study showed that happier residents had better self-rated health than those who were unhappier. Moreover, happiness could serve as a partial mediator in the effects of socioeconomic factors, social relationships factors, and age on health. Considering that both happiness and health are important indicators for a prosperous society, our studies has provided further evidence for policymakers and stakeholders focusing on happiness as a health strategy to improve overall societal wellbeing.

## Figures and Tables

**Table 1:** Variable descriptions in the model (*n* = 11,009). Abbreviations: basic medical insurance (BHI), personal commercial medical insurance (PCMI), standard deviation (SD).

Variables	Variable Descriptions	Mean (SD)
Self-rated health	Very unhealthy = 1, unhealthy = 2, normal = 3, healthy = 4, very healthy = 5	3.46 (1.10)
Happiness	Very unhappy = 1, unhappy = 2, normal = 3, happy = 4, very happy = 5	3.85 (0.85)
*demographic variables*		
Male	Male = 1, female = 0	——
Age	Continuous variable	49.97 (16.50)
Unmarried	Unmarried = 1, else = 0	——
Education	Unattained = 1, primary education = 2, middle school = 3, college and above = 4	2.73 (0.90)
*Socioeconomic factors*		
Household income	Continuous variable (natural logarithm of annual household income)	10.36 (2.06)
Individual social class	The lowest level = 1, the highest level = 5	2.36 (0.87)
Family economic level at the local	The lowest level = 1, the highest level = 5	2.54 (0.75)
*physical exercise and social relationships*		
Physical exercise	Never = 1, several times a year or less = 2, several times a month = 3, several times a week = 4, every day = 5	2.49 (1.59)
Friend interaction	Never = 1, several times a year or less = 2, several times a month = 3, several times a week = 4, every day = 5	2.37 (1.01)
Meeting relatives	Never = 1, several times a year = 2, several times a month = 3, several times a week = 4, every day = 5	2.16 (0.74)
*Insurance plan*		
BHI	Participation = 1, nonparticipation or else = 0	——
PCMI	Participation = 1, nonparticipation or else = 0	——
*Locations*		
Rural resident	Rural = 1, urban = 0	——
Province dummy variables	Dummy indicators included 28 provinces, municipalities, and autonomous regions of China and are not described here	——

**Table 2:** Descriptive statistics of different variables (*n* = 11,009). Abbreviations: basic health insurance (BHI), personal commercial medical insurance (PCMI), standard deviation (SD).

Variables		Distribution	Self-Rated Health
		(Percentage)	Mean (SD)
Self-rated health	Unhealthy (1–3)	5129 (46.6%)	-
	Healthy (4–5)	5880 (53.4%)	-
Happiness	Unhappy (1–3)	2443 (22.2%)	3.04 (1.12)
	Happy (4–5)	8566 (77.8%)	3.58 (1.06)
	*p* value		<0.001
Gender	Male	5244 (47.6%)	3.54 (1.09)
	Female	5765 (52.4%)	3.39 (1.10)
	*p* value		<0.001
Age	<45 years old	3879 (35.2%)	3.98 (0.91)
	45–59 years old	3373 (30.6%)	3.40 (1.06)
	60–74 years old	2892 (26.3%)	3.01 (1.07)
	>74 years old	865 (7.9%)	2.91 (1.06)
	*p* value		<0.001
Marital status	Unmarried	1244 (11.3%)	3.96 (0.99)
	Else	9765 (88.7%)	3.40 (1.09)
	*p* value		<0.001
Education	Unattained	1295 (11.8%)	2.84 (1.11)
	Primary education	2481 (22.5%)	3.10 (1.11)
	Middle school	5119 (46.5%)	3.58 (1.04)
	College and above	2114 (19.2%)	4.01 (0.85)
	*p* value		<0.001
Income	1st quartile	3109 (28.2%)	2.94 (1.14)
	2nd quartile	3072 (27.9%)	3.48 (1.06)
	3rd quartile	2110 (19.2%)	3.62 (0.99)
	4th quartile	2718 (24.7%)	3.93 (0.90)
	*p* value		<0.001
Social class	Below (1–2)	5748 (52.2%)	3.26 (1.12)
	Average (3)	4587 (41.7%)	3.68 (1.03)
	Above (4–5)	674 (6.1%)	3.76 (0.99)
	*p* value		<0.001
Economic level	Below (1–2)	4947 (44.9%)	3.18 (1.14)
	Average (3)	5290 (48.1%)	3.68 (1.00)
	Above (4–5)	772 (7.0%)	3.84 (0.98)
	*p* value		<0.001
Physical exercise	Infrequently (1–3)	7216 (65.5%)	3.34 (1.12)
	Frequently (4–5)	3793 (34.5%)	3.71 (1.01)
	*p* value		<0.001
Friend interaction	Infrequently (1–3)	9473 (86.0%)	3.43 (1.10)
	Frequently (4–5)	1536 (14.0%)	3.70 (1.08)
	*p* value		<0.001
Meeting relatives	Infrequently (1–3)	10,388 (94.4%)	3.45 (1.10)
	Frequently (4–5)	621 (5.6%)	3.62 (1.09)
	*p* value		<0.001
BHI	Participation	10,189 (92.6%)	3.46 (1.09)
	Not participation	820 (7.4%)	3.51 (1.14)
	*p* value		0.259
PCMI	Participation	1263 (11.5%)	3.88 (0.92)
	Not participation	9746 (88.5%)	3.41 (1.11)
	*p* value		<0.001
Rural resident	Rural	5989 (54.4%)	3.35 (1.15)
	Urban	5020 (45.6%)	3.60 (1.01)
	*p* value		<0.001

**Table 3:** Full sample regression results. Dependent variable: self-rated health.

Variables	Model 1	Model 2	Model 3	Model 4
	Coefficient (SE)	Coefficient (SE)	Coefficient (SE)	Coefficient (SE)
Happiness	0.315 *** (0.012)	0.252 *** (0.013)	0.242 *** (0.013)	0.243 *** (0.013)
Age	−0.027 *** (0.001)	−0.026 *** (0.001)	−0.026 *** (0.001)	−0.026 *** (0.001)
Male	0.159 *** (0.021)	0.170 *** (0.021)	0.164 *** (0.021)	0.164 *** (0.021)
Unmarried	0.005 (0.037)	0.040 (0.037)	0.014 (0.037)	0.008 (0.037)
Education	0.137 *** (0.015)	0.084 *** (0.016)	0.065 *** (0.016)	0.068 ***(0.016)
Income	——	0.045 *** (0.006)	0.042 *** (0.006)	0.043 *** (0.006)
Social class	——	0.106 *** (0.014)	0.101 *** (0.014)	0.101 *** (0.014)
Economic level	——	0.130 *** (0.016)	0.118 *** (0.016)	0.121 *** (0.017)
Physical exercise	——	——	0.050 *** (0.007)	0.051 *** (0.007)
Friend interaction	——	——	0.062 *** (0.012)	0.062 *** (0.012)
Meeting relatives	——	——	0.015 (0.015)	0.016 (0.015)
BHI	——	——	——	−0.130 *** (0.040)
PCMI	——	——	——	−0.005 (0.034)
Rural resident	−0.109 *** (0.026)	−0.049 * (0.026)	−0.002 (0.026)	−0.001 (0.026)
Province dummy variables	Yes	Yes	Yes	Yes
Prob > Chi2	0.000	0.000	0.000	0.000
Pseudo R2	0.106	0.116	0.119	0.119
*n*	11,009	11,009	11,009	11,009

Notes: Standard errors are given in parentheses. * *p* < 0.1. *** *p* < 0.01. All regressions include a full set of province dummy variables. Abbreviations: basic health insurance (BHI), personal commercial medical insurance (PCMI), standard errors (SE).

**Table 4:** Marginal effects of happiness on self-rated health.

Self-Rated Health	Marginal Effects
Very unhealthy	−0.019 *** (0.001)
Unhealthy	−0.038 *** (0.002)
Normal	−0.023 *** (0.001)
Healthy	0.026 *** (0.001)
Very healthy	0.053 *** (0.003)
Other variables	Yes
Province dummy variables	Yes

Notes: Average marginal effects; standard errors are given in parentheses. *** *p* < 0.01.

**Table 5:** Ordered probit regression results by age and resident type. Dependent variable: self-rated health.

Variables	The Young	The Old	Urban	Rural
	Coefficient (SE)	Coefficient (SE)	Coefficient (SE)	Coefficient (SE)
Happiness	0.247 *** (0.016)	0.230 *** (0.023)	0.270 *** (0.021)	0.230 *** (0.017)
Age	−0.029 *** (0.001)	−0.011 *** (0.003)	−0.026 *** (0.001)	−0.025 *** (0.001)
Male	0.150 *** (0.026)	0.214 *** (0.036)	0.155 *** (0.031)	0.175 *** (0.029)
Unmarried	−0.036 (0.044)	0.035 (0.094)	−0.030 (0.053)	0.051 (0.053)
Education	0.069 *** (0.021)	0.054 ** (0.025)	0.030 (0.025)	0.092 *** (0.021)
Income	0.050 *** (0.008)	0.033 *** (0.008)	0.017 (0.011)	0.047 *** (0.007)
Social class	0.111 *** (0.017)	0.083 *** (0.023)	0.075 *** (0.022)	0.118 *** (0.018)
Economic level	0.121 *** (0.021)	0.103 *** (0.027)	0.128 *** (0.025)	0.126 *** (0.022)
Physical exercise	0.039 *** (0.009)	0.077 *** (0.012)	0.070 *** (0.010)	0.028 *** (0.010)
Friend interaction	0.070 *** (0.016)	0.053 *** (0.018)	0.068 *** (0.018)	0.054 *** (0.016)
Meeting relatives	0.018 (0.020)	0.012 (0.024)	0.007 (0.022)	0.020 (0.022)
BHI	−0.103 ** (0.049)	−0.173 ** (0.069)	−0.071 (0.062)	−0.156 *** (0.052)
PCMI	−0.004 (0.038)	0.021 (0.086)	0.032 (0.043)	−0.030 (0.060)
Rural resident	0.013 (0.032)	−0.018 (0.049)	——	——
Prob > Chi2	0.000	0.000	0.000	0.000
Pseudo R2	0.102	0.069	0.108	0.125
*n*	7252	3757	5020	5989

Notes: Standard errors are given in parentheses. ** *p* < 0.05. *** *p* < 0.01. All regressions include a full set of province dummy variables. Abbreviation: basic health insurance (BHI), personal commercial medical insurance (PCMI).

**Table 6:** Marginal effects of happiness on self-rated health.

	The Young	The Old	Urban	Rural
Self-rated health	Marginal effects	Marginal effects	Marginal effects	Marginal effects
Very unhealthy	−0.012 *** (0.001)	−0.031 *** (0.003)	−0.014 *** (0.001)	−0.023 *** (0.002)
Unhealthy	−0.032 *** (0.002)	−0.045 *** (0.005)	−0.037 *** (0.003)	−0.037 *** (0.003)
Normal	−0.035 *** (0.002)	−0.001 (0.001)	−0.038 *** (0.003)	−0.014 *** (0.001)
Healthy	0.013 *** (0.001)	0.049 *** (0.005)	0.026 *** (0.002)	0.027 *** (0.002)
Very healthy	0.066 *** (0.004)	0.027 *** (0.003)	0.063 *** (0.005)	0.048 *** (0.004)
Other variables	Yes	Yes	Yes	Yes
Province dummy variables	Yes	Yes	Yes	Yes

Notes: Average marginal effects, standard errors are given in parentheses. *** *p* < 0.01.

**Table 7:** Results of the robustness check.

Variables	IVoprobit	OLS	Probit
	Coefficient (SE)	Coefficient (SE)	Coefficient (SE)
Happiness	0.744 ** (0.322)	0.210 *** (0.011)	0.242 *** (0.017)
Age	0.027 *** (0.002)	−0.022 *** (0.001)	−0.025 *** (0.001)
Male	0.172 *** (0.0250)	0.140 *** (0.018)	0.165 *** (0.027)
Unmarried	−0.032 (0.080)	−0.026 (0.031)	0.056 (0.049)
Education	0.049 (0.032)	0.065 *** (0.014)	0.080 *** (0.020)
Income	0.042 *** (0.011)	0.039 *** (0.005)	0.033 *** (0.008)
Social class	0.011 (0.063)	0.089 *** (0.012)	0.130 *** (0.018)
Economic level	0.011 (0.088)	0.107 *** (0.014)	0.122 *** (0.021)
Physical exercise	0.028 (0.017)	0.043 *** (0.006)	0.041 *** (0.009)
Friend interaction	0.060 *** (0.017)	0.053 *** (0.010)	0.077 *** (0.015)
Meeting relatives	−0.028 (0.032)	0.015 (0.013)	−0.012 (0.020)
BHI	0.124 *** (0.047)	−0.105 *** (0.034)	−0.122 ** (0.051)
PCMI	0.012 (0.042)	−0.006 (0.029)	0.029 (0.044)
Rural resident	−0.002 (0.033)	−0.004 (0.023)	−0.006 (0.034)
Province dummy variables	Yes	Yes	Yes
Prob > Chi2	0.000	0.000	0.000
R2	-	0.297	0.170
*n*	8458	11,009	11,009
F-statistic	11.07	-	-

Notes: Standard errors are given in parentheses. ** *p* < 0.05. *** *p* < 0.01. Abbreviation: basic health insurance (BHI), personal commercial medical insurance (PCMI).

**Table 8:** Structural model fit indexes.

Model Fit	χ^2^/DF (*p*)	IFI	TLI	AGFI	CFI	RMSEA
Model	27.28 (<0.05)	0.981	0.949	0.980	0.981	0.049
Cut-off criteria	≤3 (≥0.05)	≥0.90	≥0.90	≥0.90	≥0.90	≤0.08

Note: Cut-off criteria according to reference [59,60]. Abbreviation: chi-square divided by degrees of freedom (χ^2^/DF), Tucker–Lewis index (TLI), comparative fit index (CFI), root mean square error of approximation (RMSEA), and incremental fit index (IFI).

**Table 9:** Path coefficients of structural model. Abbreviations: standard errors (SE).

			b	SE	β	*p* Value
Happiness	←	Social relationship	0.058	0.019	0.043	0.002
Happiness	←	Social economic	0.647	0.023	0.414	0.000
Happiness	←	Age	0.003	0.000	0.049	0.000
Self-rated health	←	Social economic	0.448	0.027	0.226	0.000
Self-rated health	←	Social relationship	0.187	0.022	0.107	0.000
Self-rated health	←	Age	−0.025	0.001	−0.375	0.000
Self-rated health	←	Happiness	0.168	0.013	0.132	0.000

Notes: b denotes the coefficient of corresponding paths; β represents the standardized coefficient of corresponding paths.

## Data Availability

The raw data used in this study are from the Chinese General Social Survey (http://cnsda.ruc.edu.cn/index.php?r=projects/view&id=62072446) (accessed on 3 September 2021), and unrestricted reuse is permitted.

## References

[1] Yip W., Fu H., Chen A.T., Zhai T., Jian W., Xu R., Pan J., Hu M., Zhou Z., Chen Q. (2019). 10 years of health-care reform in China: Progress and gaps in Universal Health Coverage. Lancet.

[2] Wang M., Yang Y., Jin S., Gu L., Zhang H. (2016). Social and cultural factors that influence residential location choice of urban senior citizens in China—The case of Chengdu city. Habitat Int..

[3] Diener E., Heintzelman S.J., Kushlev K., Tay L., Wirtz D., Lutes L.D., Oishi S. (2017). Findings all psychologists should know from the new science on subjective well-being. Can. Psychol. Can..

[4] Diener E., Oishi S., Lucas R.E. (2015). National accounts of subjective well-being. Am. Psychol..

[5] Boehm J.K., Lyubomirsky S., Sheldon K.M. (2011). A longitudinal experimental study comparing the effectiveness of happiness-enhancing strategies in Anglo Americans and Asian Americans. Cogn. Emot..

[6] Emmons R.A., McCullough M.E. (2003). Counting blessings versus burdens: An experimental investigation of gratitude and subjective well-being in daily life. J. Pers. Soc. Psychol..

[7] Lyubomirsky S., Dickerhoof R., Boehm J.K., Sheldon K.M. (2011). Becoming happier takes both a will and a proper way: An experimental longitudinal intervention to boost well-being. Emotion.

[8] Lyubomirsky S., King L., Diener E. (2005). The Benefits of Frequent Positive Affect: Does Happiness Lead to Success?. Psychol. Bull..

[9] Seligman M.E.P., Steen T.A., Park N., Peterson C. (2005). Positive Psychology Progress: Empirical Validation of Interventions. Am. Psychol..

[10] King L.A. (2001). The Health Benefits of Writing about Life Goals. Pers. Soc. Psychol. Bull..

[11] Layous K., Nelson S.K., Lyubomirsky S. (2013). What Is the Optimal Way to Deliver a Positive Activity Intervention? The Case of Writing About One’s Best Possible Selves. J. Happiness Stud..

[12] Pinquart M., Forstmeier S. (2012). Effects of reminiscence interventions on psychosocial outcomes: A meta-analysis. Aging Ment. Health.

[13] Lambert N.M., Gwinn A.M., Baumeister R., Strachman A., Washburn I.J., Gable S.L., Fincham F. (2012). A boost of positive affect. J. Soc. Pers. Relatsh..

[14] Danner D.D., Snowdon D.A., Friesen W.V. (2001). Positive emotions in early life and longevity: Findings from the nun study. J. Pers. Soc. Psychol..

[15] Diener E., Oishi S., Tay L. (2018). Advances in subjective well-being research. Nat. Hum. Behav..

[16] Pressman S.D., Cohen S. (2012). Positive emotion word use and longevity in famous deceased psychologists. Health Psychol..

[17] Weiss A., Adams M.J., King J.E. (2011). Happy orang-utans live longer lives. Biol. Lett..

[18] Sadler M.E., Miller C.J., Christensen K., McGue M. (2011). Subjective Wellbeing and Longevity: A Co-Twin Control Study. Twin Res. Hum. Genet..

[19] Chida Y., Steptoe A. (2008). Positive Psychological Well-Being and Mortality: A Quantitative Review of Prospective Observational Studies. Psychosom. Med..

[20] Davidson K.W., Mostofsky E., Whang W. (2010). Don’t worry, be happy: Positive affect and reduced 10-year incident coronary heart disease: The Canadian Nova Scotia Health Survey. Eur. Heart. J..

[21] Sabatini F. (2014). The relationship between happiness and health: Evidence from Italy. Soc. Sci. Med..

[22] Liu B., Floud S., Pirie K., Green J., Peto R., Beral V. (2016). Does happiness itself directly affect mortality? The prospective UK Million Women Study. Lancet.

[23] Frey B.S. (2011). Happy People Live Longer. Science.

[24] Cuijpers P., Smit F. (2002). Excess mortality in depression: A meta-analysis of community studies. J. Affect. Disord..

[25] Golden S.H., Williams J.E., Ford D.E., Yeh H.C., Paton S.C., Nieto F.J., Brancati F.L. (2004). Depressive symptoms and the risk of type 2 diabetes: The Atherosclerosis Risk in Communities study. Diabetes Care.

[26] Hemingway H., Marmot M. (1999). Evidence based cardiology: Psychosocial factors in the aetiology and prognosis of coronary heart disease: Systematic review of prospective cohort studies. BMJ.

[27] Penninx B., Leveille S., Ferrucci L., Van Eijk J., Guralnik J. (1999). Exploring the effect of depression on physical disability: Longitudinal evidence from the established populations for epidemiologic studies of the elderly. Am. J. Public Health.

[28] Ryan R.M., Deci E.L. (2001). On Happiness and Human Potentials: A Review of Research on Hedonic and Eudaimonic Well-Being. Annu. Rev. Psychol..

[29] Suls J., Bunde J. (2005). Anger, Anxiety, and Depression as Risk Factors for Cardiovascular Disease: The Problems and Implications of Overlapping Affective Dispositions. Psychol. Bull..

[30] James G.D., Yee L.S., A Harshfield G., Blank S.G., Pickering T.G. (1986). The influence of happiness, anger, and anxiety on the blood pressure of borderline hypertensives. Psychosom. Med..

[31] Pressman S.D., Cohen S. (2005). Does positive affect influence health?. Psychol. Bull..

[32] Schnall P.L., Pieper C., Schwartz J.E., Karasek R.A., Schlussel Y., Devereux R.B. (1990). The relationship between ‘job strain,’ workplace diastolic blood pressure, and left ventricular mass index. Results of a case-control study. J. Am. Med. Asso..

[33] Barak Y. (2006). The immune system and happiness. Autoimmun. Rev..

[34] Rasciute S., Downward P. (2010). Health or Happiness? What Is the Impact of Physical Activity on the Individual?. Kyklos.

[35] Veenhoven R. (2008). Healthy happiness: Effects of happiness on physical health and the consequences for preventive health care. J. Happiness Stud..

[36] Malmusi D., Vives A., Benach J., Borrell C. (2014). Gender inequalities in health: Exploring the contribution of living conditions in the intersection of social class. Glob. Health Action.

[37] Wang F.Q. (2012). Socioeconomic status, lifestyle and health inequality. Chin. J. Sociol..

[38] Subramanian S.V., Kim D.J., Kawachi I. (2002). Social trust and self-rated health in US communities: A multilevel analysis. J. Urban Health.

[39] Al-Shami N.m.A., Shojaia H., Darwish H., Giacaman R. (2017). Factors associated with self-rated health among elderly Palestinian women: An analysis of cross-sectional survey data. Lancet.

[40] Ferrer-i-Carbonell A. (2005). Income and well-being: An empirical analysis of the comparison income effect. J. Public Econ..

[41] Luttmer E. (2005). Neighbors as Negatives: Relative Earnings and Well-Being. Q. J. Econ..

[42] Jebb A.T., Tay L., Diener E., Oishi S. (2018). Happiness, income satiation and turning points around the world. Nat. Hum. Behav..

[43] Borghesi S., Vercelli A. (2012). Happiness and health: Two paradoxes. J. Econ. Surv..

[44] Wilkinson R.G. (1992). Income distribution and life expectancy. BMJ.

[45] Deaton A. (2003). Health, Inequality, and Economic Development. J. Econ. Lit..

[46] Holt-Lunstad J., Smith T.B., Layton J.B. (2010). Social Relationships and Mortality Risk: A Meta-analytic Review. PLoS Med..

[47] Lim C., Putnam R.D. (2010). Religion, Social Networks, and Life Satisfaction. Am. Sociol. Rev..

[48] Pichler F. (2006). Subjective Quality of Life of Young Europeans. Feeling Happy but who Knows why?. Soc. Indic. Res..

[49] Barth J., Schneider S., von Känel R. (2010). Lack of social support in the etiology and the prognosis of coronary heart disease: A systematic review and meta-analysis. Psychosom. Med..

[50] Hakulinen C., Pulkki-Råback L., Jokela M., Ferrie J.E., Aalto A.M., Virtanen M., Kivimäki M., Vahtera J., Elovainio M. (2016). Structural and functional aspects of social support as predictors of mental and physical health trajectories: Whitehall II cohort study. J. Epidemiol. Community Health.

[51] DeSalvo K.B., Bloser N., Reynolds K., He J., Muntner P. (2006). Mortality prediction with a single general self-rated health question. A meta-analysis. J. Gen. Intern. Med..

[52] Jylhä M. (2009). What is self-rated health and why does it predict mortality? Towards a unified conceptual model. Soc. Sci. Med..

[53] Rütten A., Abel T., Kannas L., Von Lengerke T., Lüschen G., Rodriguez Diaz J.A., Vinck J., Van der Zee J. (2001). Self reported physical activity, public health, and perceived environment: Results from a comparative European study. J. Epidemiol. Community Health.

[54] Diener E. (1984). Subjective well-being. Psychol. Bull..

[55] Frey B.S., Stutzer A. (2002). What Can Economists Learn From Happiness Research. J. Econ. Lit..

[56] Asadullah M.N., Xiao S., Yeoh E. (2018). Subjective well-being in China, 2005–2010: The role of relative income, gender, and location. China Econ. Rev..

[57] Steptoe A., Deaton A., Stone A.A. (2015). Subjective wellbeing, health, and ageing. Lancet.

[58] Chen H., Liu Y., Zhu Z., Li Z. (2017). Does where you live matter to your health? Investigating factors that influence the self-rated health of urban and rural Chinese residents: Evidence drawn from Chinese General Social Survey data. Health Qual. Life Outcomes.

[59] Maridal J.H. (2016). A Worldwide Measure of Societal Quality of Life. Soc. Indic. Res..

[60] Hu L., Bentler P.M. (1999). Cut-Off Criteria for Fit Indexes in Covariance Structure Analysis: Conventional Criteria versus New Alternatives. Struct. Equ. Modeling.

[61] Gong P., Liang S., Carlton E.J., Jiang Q., Wu J., Wang L., Remais J.V. (2012). Urbanisation and health in China. Lancet.

[62] Li X., Song J., Lin T., Dixon J., Zhang G., Ye H. (2016). Urbanization and health in China, thinking at the national, local and individual levels. Environ. Health.

[63] Miao J., Wu X. (2016). Urbanization, socioeconomic status and health disparity in China. Health Place.

[64] Zhu Y.G., Ioannidis J.P., Li H., Jones K.C., Martin F.L. (2011). Understanding and Harnessing the Health Effects of Rapid Urbanization in China. Environ. Sci. Technol..

